# Assessment of Heavy Metal Contamination and Health Risks in “Snow Cover–Soil Cover–Vegetation System” of Urban and Rural Gardens of an Industrial City in Kazakhstan

**DOI:** 10.3390/ijerph21081002

**Published:** 2024-07-30

**Authors:** Alina Faurat, Galymbek Azhayev, Kazbek Shupshibayev, Kairat Akhmetov, Elmira Boribay, Talgat Abylkhassanov

**Affiliations:** 1 Department of Geography and Tourism, Toraighyrov University, Pavlodar 140008, Kazakhstan; agalymbek@mail.ru; 2 Department of Ecology, Saken Seifullin Kazakh Agrotechnical University, Astana 010011, Kazakhstan; kazbek_61@mail.ru; 3 Department of Biology and Ecology, Toraighyrov University, Pavlodar 140008, Kazakhstan; kairat_akhmetov@mail.ru (K.A.); talgat.abylkhassanov@gmail.com (T.A.); 4 Ecology Educational Program, Narxoz University, Almaty 050060, Kazakhstan; elmira.boribay@narxoz.kz

**Keywords:** heavy metals, metalloids, vegetable contamination, pollution indices, health risk assessment

## Abstract

This article investigates the extent of heavy metal pollution in both urban and rural gardens in Pavlodar, which cultivate potatoes and tomatoes. As a city of industrialization, Pavlodar is exposed to emissions from industrial enterprises, transport and stove heating. The city also has the highest incidence of environmental diseases among the population. This study examines the accumulation of heavy metals and metalloid in the snow, their migration into the soil and their accumulation in plants, and assesses the non-cancer and cancer health risks of consuming these vegetables. The results show that the concentrations of trace elements in the solid phase of snow decrease in the following order: Fe (26,000) > Mn (592.5) > Cr (371.3) > Zn (338.8) > Pb (161.9) > Cu (142.5) > Ni (30.9) > As (15.1) > Co (12.1) > Cd (2.6). In soils, the concentrations of elements decrease in the following order: Mn (22,125) > Fe (20,375) > Zn (246.9) > Cr (109.5) > Cu (39.3) > Pb (25.6) > Ni (22.4) > As (9) > Co (6.6) > Cd (0.2). In urban gardens, the snow pollution coefficient was the highest. In rural gardens, the contamination index varied from 0.3 (Cr) to 5.3 (Cd). Magnesium in the soil exceeds the maximum allowable concentration (MPC) by 28.6–35.7 times, and zinc by 1.6–10.9 times. Only zinc and copper exceed the MPC for vegetables. Nickel in potatoes exceeds MPC by a factor of 6 and in tomatoes by a factor of 4.4. The cobalt content in tomatoes exceeds the background value by 2.2 times, with a maximum value of 5.3 times. The risk assessment showed that the non-carcinogenic and carcinogenic risks associated with potato and tomato consumption were low. However, these risks are higher in urban areas than in rural areas.

## 1. Introduction

The migration of heavy metals from snow to soil and plants is a serious environmental concern because of its potential impacts on ecosystems and human health. The accumulation of heavy metals in soil can lead to the deterioration of soil quality, reduced crop yields and poor quality of agricultural products, posing risks to human health and the environment [1].

Snow cover is capable of accumulating almost all substances that enter the atmosphere from various sources, making it a convenient indicator of pollution for monitoring urban air and soil pollution. Studies have shown that melt water and dust settling on the snow surface contain significant amounts of heavy metals, making snow a stable environment for monitoring heavy metal pollution in the atmosphere [2,3].

Factors such as the total accumulation of heavy metals in soil, soil pH and soil organic matter content play important roles in the transfer of heavy metals from soil to plants [4]. The adsorption and deposition of heavy metals in the topsoil are critical factors affecting the migration of heavy metals [2,5].

Understanding the migration of heavy metals in soil profiles is essential for effective pollution control and environmental management. Studies of heavy metal movements under specific climatic conditions can provide valuable information for pollution control strategies [6].

First and foremost, environmental monitoring should be conducted to assess the degree of pollutant accumulation in different environments in order to prevent their negative impact on human health. Heavy metals and metalloids are particularly harmful because they are persistent, bioaccumulative and toxic (PBT) pollutants [7]. The biological activity of heavy metals/metalloids places this group of pollutants at the forefront of environmental monitoring studies. Heavy metals are known to have carcinogenic, mutagenic and teratogenic properties. They cause the formation of reactive oxygen species, leading to oxidative stress and diseases, and act as metabolic poisons, inhibiting enzymes of the sulphhydryl systems involved in the production of cellular energy [8]. 

The most dangerous heavy metals and metalloids for the environment are Cr, Ni, Cu, Zn, Cd, Pb, Hg and As, which are included in the list of elements to be controlled [9]. Arsenic (As), cadmium (Cd) and lead (Pb) are among the top ten chemicals of public health concern for WHO [10]. 

The adverse effects of heavy metals and metalloids have been linked to effects on the nervous system, endocrine disruption, reproductive and developmental problems, cancer and genetic abnormalities [7]. Childhood exposure to lead and mercury can impair cognitive, neurological and developmental functions. Arsenic and cadmium increase cancer risk with long-term exposure, and inhalation of lead and cadmium contributes to respiratory and cardiovascular disease [11] as well as affecting the human immune system, which plays a key role in the pathophysiology of cancer [12]. Food and water contaminated with heavy metals cause gastrointestinal problems and damage the digestive system [11]. These elements’ trophic transfer in food chains is critical for human health. Concentrations of potentially toxic heavy metals and metalloids need to be assessed and monitored in various segments of the environment and local biota, especially in vegetables grown in cities and large urban centres, which serve as a source of food for the local population [13].

Urban gardens are necessary to provide fresh food to city residents and suburban communities. However, the presence of contaminants such as heavy metals and organic chemicals in urban soils raises concerns about potential health risks [14]. Factors such as proximity to roads and industrial activities contribute to the accumulation of heavy metals such as lead, cadmium and metalloid arsenic in urban garden soils [15,16,17,18]. 

More than 60.3 garden plots and private vegetable gardens grow vegetables, potatoes, fruits and berries on the territory of the city of Pavlodar (Kazakhstan) [19]. The city of Pavlodar is one of the largest industrial centres in the Republic of Kazakhstan. The northern and eastern districts of the city are complex and include industrial areas. The northern industrial district has metallurgical industry (ferrous, non-ferrous), power industry (CHPP-2 (Combined Heat and Power Producing), CHPP-3), chemical industry (Pavlodar Petrochemical Plant, JSC “Kaustik”), engineering and other branches of production. In the eastern zone are located JSC “Aluminium of Kazakhstan”, JSC “Kazakhstan Electrolysis Plant”, JSC “Pavlodar Machine-Building Plant” and CHPP-1. Enterprises in the fuel and energy complex contribute the most to the city’s pollution, accounting for 66.2%, followed by metallurgy (23.5%), petrochemicals (3.2%), mining (1.7%) and other industries (5.4%). The Pavlodar region is Kazakhstan’s leader in terms of environmental emissions. In 2022, emissions of pollutants into the atmosphere from stationary sources amounted to 724.2 thousand tonnes [20]. 

Studies on the morbidity of the population in the Republic of Kazakhstan revealed that the Pavlodar region stands out for having higher adult morbidity rates for all pathologies caused by the environment. In children in the Pavlodar region, the indices of the majority of pathologies exceed the average republican level by 1.5–3.9 times. The city of Pavlodar has the highest indicators of respiratory diseases and malignant neoplasms among both adults and children. The region is classified as an area with a challenging medical and environmental situation [21,22]. 

It is necessary to monitor the concentrations of potentially toxic metals and metalloids in various environmental media that affect the city’s public health. A systemic picture will be obtained by studying the accumulation of pollutants: in the snow cover, which also provides information on atmospheric air pollution [23]; in the soil cover and the transfer of pollutants to vegetables eaten by the local population. This systematic approach will provide useful information on the main sources, distribution and migration of these elements in the environment, as well as their bioaccumulation in food chains.

Therefore, the aim of our study was to investigate heavy metal contamination in the snow–soil–vegetable system in urban and rural gardens. Within the framework of the task, it is necessary to solve the following tasks: Determine the content of heavy metals in the snow cover, soil and vegetables of the city of Pavlodar (1); Identify the patterns of spatial distribution of microelements in the studied environments (urban and rural gardens) (2); Assess the degree of contamination of snow and soil with heavy metals/metalloid using pollution indices and standards (3); Calculate the bioaccumulation of heavy metals/ metalloid from the soil in potatoes and tomatoes grown in urban and rural gardens (4); Calculate carcinogenic and non-carcinogenic risk indices for the health of people consuming the grown products (5).

## 2. Materials and Methods

### 2.1. Study Area and Sample Collection

Pavlodar is situated in Kazakhstan’s north-east. The city is situated on the first alluvial terrace of the Irtysh River, which gradually becomes a lacustrine-eluvial plain to the east of the city. The soils belong to the chestnut soil zone; their mechanical composition is light loamy. Chestnut soils, characteristic of the dry steppe zone, have a humus horizon (A + B) 40–50 cm thick with a humus content of 3.5–4.0%. These soils are associated with solonets and show signs of solonetz [24].

**Figure 1:**
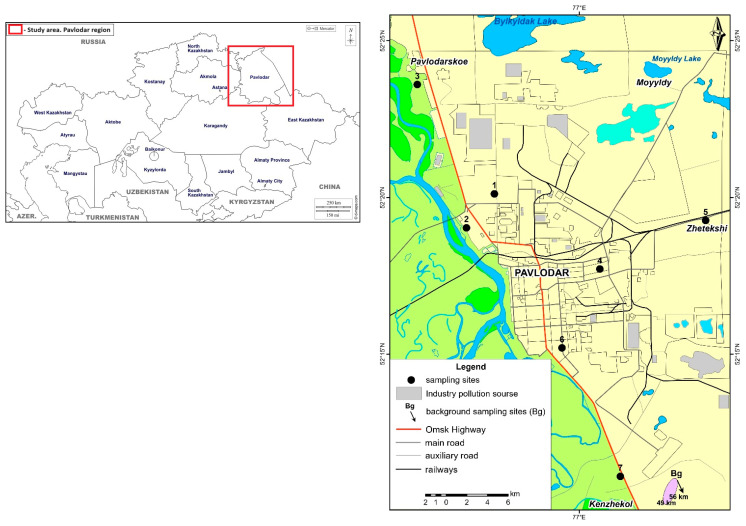
Location of the study area and sampling sites in the city of Pavlodar and its suburbs. Sites 1, 2, 4, 6—urban garden; Sites 3, 5, 7—rural gardens.

Sampling was carried out in 2023 in different areas of the city according to standard methodological recommendations [23]. Seven discrete sampling points were identified: sites 1, 2, 4 and 6 in urban gardens; sites 3, 5 and 7 in rural gardens (Figure 1). Snow and soil sampling were carried out using the envelope method. At each site, points were selected to form a rectangle with a point in the centre (5-point samples). The side of the rectangle for snow and soil was 5 m. For vegetables, at least 8-point samples were selected. A pooled sample was made by mixing and taking the required amount (weight) of the point samples. Therefore, we collected one snow cover sample, one soil sample, one tomato sample and one potato sample from each of the seven sites [25].

Soil, snow and vegetable samples were collected from the plots of vegetable gardens and dachas located within the city limits and in the nearest settlements to the city of Pavlodar.

Snow sampling was conducted in January, with an average snow depth of 60 cm. The sampling method used was the pit method: the depression (depth, width, height) in the snow cover was measured to its full thickness. The snow was collected in plastic bags, and the average sample weight was 6 kg. The snow was processed under laboratory conditions and melted under natural conditions within 8–12 h. The meltwater was filtered through ash-free filters, and the filtrated water (1000 mL) was acidified with 5 mL of nitric acid. The resulting sediment was dried, weighed and packaged for further work to determine the content of heavy metals/metalloid.

After snowmelt (April), soil samples were taken at the same points as the snow cover samples. Samples were taken from 0–15 cm in depth.

Background samples were taken more than 50 km from the city boundary, in the opposite direction to the prevailing wind direction. The background samples were taken in a small settlement in a zone of chestnut soils, which also includes urban soils. Thus, the snow, soils and vegetables of the background samples were also affected by anthropogenic influences (stove heating, car exhausts, agriculture), but the influence of urban industry and active exposure from motor vehicles is limited [26,27].

The sampling points for vegetable crops coincide with the sampling points for snow cover and soil cover on private plots in the city and suburbs in order to determine the patterns of heavy metal content in different environments. The sampling of vegetable crops was carried out according to the requirements of Guidelines for the determination of heavy metals in soils of farmland and crop products, 1992 [25].

At each test point, 2 samples were taken, tomato fruits (Family: *Solanaceae*, Species: *Solanum lycopersicum* L.) and potato tubers (Family: *Solanaceae*; Species: Potato—*Solanum tuberosum* L.), to determine the accumulation of heavy metals/metalloid in the soil above ground and in the underground parts of the plants. The choice of plants was based on their widespread cultivation and importance in the urban population’s diet. A total of 16 samples were collected, each weighing 1 kg. The samples selected for chemical analysis were packaged and prepared for transport, taking into account storage in containers made of chemically neutral material.

### 2.2. Laboratory Studies

Laboratory tests were carried out in the laboratory of the branch of the Institute of Radiation Safety and Ecology of the RSE National Nuclear Center of the Republic of Kazakhstan. The analysis of the content of chemical elements in the solid phase of snow and soil was carried out by inductively coupled plasma mass spectrometry using an Agilent 7700 X ICP-MS (Agilent Technologies, Inc., Santa Clara, CA, USA) in accordance with MVI No. 499-AES/MS MCHA “Methods of quantitative chemical analysis”. Determination of elemental composition of rocks, soils and bottom sediments by atomic emission with inductively coupled plasma and mass spectrometry with inductively coupled plasma methods” (Registration number: KZ.07.00.03351-2016) [28]. The analysis of the content of chemical elements in the liquid phase of snow was carried out in accordance with GOST ISO 17294-2-2019 Quality Water [29]. The content of heavy metals in edible parts of garden crops: tomato fruits and potato roots was determined by inductively coupled plasma mass spectrometry using an Agilent 7700 X ICP-MS. Plant samples were digested by microwave digestion according to Berhof (Speedware Xpert) (BERGHOFF Products + Instruments GmbH, Eningen unter Achalm, Germany) and M-MVI-2008 guidelines, 2008 [30]. Analysis of chemical elements in water and plant samples according to the GOST ISO 17294-2019 Water Quality Method [29].

For processing, 10 elements were selected, which are the most toxic and also make a large contribution to the pollution of snow, soil cover and vegetables in the city: Cr, Mn, Fe, Co, Ni, Cu, Zn, As, Cd and Pb. The selection of these metals is based on the list of heavy metals and toxic elements included in the international and national lists of pollutants subject to control (“Methodological recommendations…”, 1982). All these metals, except for iron, are classified into three hazard classes: Zn, As, Cd and Pb—class 1; Cr, Co, Ni and Cu—class 2; Mn—class 3 (GOST 17.4.1.02-83) [31]. The inclusion of iron in the list of metals under consideration is due to the high concentrations of the element in snow and soil cover. Arsenic (As), cadmium (Cd) and lead (Pb) are among the top ten chemicals of public health concern [10].

### 2.3. Contamination Assessment

In order to assess the degree of heavy metal contamination of soils and plants, indices have been used to provide a unified assessment of soil contamination with individual elements. These include the pollution index relative to the background content of heavy metals/metalloid obtained in our studies—PI; the enrichment coefficient relative to the clark concentration in soils—EF; the exceeding of the maximum allowable concentration (MAC), the coefficient of bioaccumulation of heavy metals/metalloid in plants or the factor of transfer of microelements from soil to plants—BAC (TF).

The health risk assessment included the calculation of the daily intake (CDI), the individual metal hazard index (HQ), the hazard or non-carcinogenic risk index (HI), the individual metal carcinogenic risk (CR) and the cumulative carcinogenic risk (LCR) [32].

#### 2.3.1. Pollution Indices

The single pollution index (PI) is often used to identify the most dangerous heavy metals for the soil environment.

Pollution Index (PI)

(1) $$PI=\frac{Cn}{Bg},$$

where Cn is the heavy metal content of the soil and Bg is the heavy metal content of the background samples.

Enrichment Factor EF

The EF coefficient is a measure of the possible influence of anthropogenic activities on the concentration of elements in soil [33,34]:

(2) $$EF=\frac{\frac{Cn}{Fe}sample}{\frac{Cn}{Fe}background},$$

where Cn sample is the concentration of the metal under investigation and Fe is the concentration of iron used as a reference in this study.

An exceedance of the MAC (Maximum Allowable Concentration) occurs when the concentration of a contaminant in the environment (soil, plants, etc.) exceeds the level allowed by regulations [19]:

(3) $$MPC\;exceedance=\frac{Cn}{MPC},\;$$

where Cn is the concentration of pollutant in soil or plant; MPC is maximum allowable concentration.

Coefficient of heavy metal accumulation in soil (BAC) (transfer factor, TF):

(4) $$BAC\left(TF\right)=\frac{Csoil}{Csnow},$$

where Csoil is the concentration of heavy metal in soil; Csnow is the heavy metal concentration in snow.

#### 2.3.2. Human Health Risk Assessment

Health risk assessment indices are widely used to quantify human exposure to chemical elements, including both carcinogenic and non-carcinogenic risks from vegetable consumption.

In this study, we apply an exposure model based on the methodology developed by the US Environmental Protection Agency [35] and presented in [32]. Formulas for calculating the chronic daily intake (CDI) of potentially toxic metals in adults and children:

(5) $$CDI=\frac{C\times ingR\times CF\times EF\times ED}{BW\times AT},$$

where C is the concentration in soil (mg/kg), R is amount of vegetable consumption: potato (0.123 mg/day (adults), 0.0615 mg/day (children); tomato (0.022 mg/day (adults), 0.011 mg/day (children), CF—10-3; EF—frequency of exposure (350 days/year); ED—exposure duration (24 years (adults), 6 years (children)); AT—exposure duration (24 h) days); BW—body weight of the exposed person (70 kg (adults), 20 kg (children)); AT—averaging time (day), 365 × ED adult/children; ET is the exposure duration (24 h/d).

The hazard index (HI) is a tool for assessing the overall potential non-cancer risk associated with human exposure to toxic metals. The hazard index (HI) calculations were performed as follows:

(6) $$HI=\sum HQ_{i},$$

(7) $$HQ_{i}=\sum \frac{ADD_{i}}{RfD_{i}},$$

where RfD (mg kg^−1^ day^−1^) is the reference dose for each heavy metal [36]. In this study, nine heavy metals with a reference dose—chromium (1.5), manganese (0.14), iron (0.7), nickel (0.02), copper (0.04), zinc (0.3) and lead (0.0035)—were considered in the context of potential non-cancer risks.

If the hazard index (HI) value is less than 1, there is probably no risk of non-cancer effects. However, if the HI is greater than 1, adverse health effects are possible, and the likelihood of these effects increases as the HI increases.

Metals such as As, Ni and Cr are classified as metals with a carcinogenic risk. The carcinogenic risk score (LCR) can be expressed as

(8) $$LCR=\sum ADD_{i}\times SF_{i},$$

SF (mg kg^−1^ day^−1^)^−1^ probability of carcinogenic risk for toxic metals [36]. The following metals with some risk were considered: chromium (0.5), nickel (0.91) and lead (0.0085). If CR <10^−6^, the carcinogenic risk is considered negligible; if CR >10^−4^, there is a high risk of a person developing cancer; and if 10^−6^ < CR < 10^−4^, there is an acceptable risk to humans [37].

### 2.4. Statistical Analysis

Means, minimums, maximums, standard deviations, coefficients of variation and all heavy metal indices were calculated and summarized in Microsoft Excel, the version number 2406 (Microsoft Inc., Redmond, WA, USA).

## 3. Results and Discussion

### 3.1. Heavy Metal Concentrations in Urban Media

The content of heavy metals/ metalloid in the liquid phase (LSS), the solid phase (SPS) of snow, the soil and the mass concentration of trace elements in vegetables are presented in Table 1. The highest concentrations of trace elements are observed in the solid phase of snow, with the following decreasing series: Fe (26,000) > Mn (592.5) > Cr (371.3) > Zn (338.8) > Pb (161.9) > Cu (142.5) > Ni (30.9) > As (15.1) > Co (12.1) > Cd (2.6). The concentration of trace elements varied significantly between samples in the following range: Cr (220–690 mg/kg), Mn (530–670 mg/kg), Fe (21,000–35,000 mg/kg), Co (8–22 mg/kg), Ni (19–49 mg/kg), Cu (40–430 mg/kg), Zn (120–590 mg/kg), As (6–37 mg/kg), Cd (1–6.1 mg/kg), Pb (20–300 mg/kg).

Pollutant concentrations in the liquid phase of snow (meltwater) were lower than in solid snow sediments and soils but higher than in vegetables, although the closest value was observed. The concentration of trace elements in meltwater was as follows, in descending order: Fe (222.8) > Mn (29.1) > Zn (28.3) > Pb (5.5) > Cu (3.5) > As (3.0) > Cr (1.7), while the concentrations of cobalt, nickel and cadmium were less than 0.1 mg/kg. In general, the decreasing series corresponds to that for solid snow sediment, but the concentration of chromium in meltwater has low values, indicating that chromium is a metal present in an insoluble form. The range of changes in metal concentrations in the liquid phase of snow is as follows: Cr (0.5–3.2 mg/kg), Mn (16–72 mg/kg), Fe (74–1000 mg/kg), Cu (1.1–8.8 mg/kg), Zn (6–47 mg/kg), As (1.4–4 mg/kg), Pb (2.4–8.9 mg/kg).

The decreasing series of the average content of heavy metals / metalloid in the soils on which vegetables are grown in the city of Pavlodar has the following form: Mn (22,125) > Fe (20,375) > Zn (246.9) > Cr (109.5) > Cu (39.3) > Pb (25.6) > Ni (22.4) > As (9) > Co (6.6) > Cd (0.2). Comparing the concentrations of elements in solid snow sediment with soil data, it was found that the metal content of snow is much higher than that of soil. For example, cadmium in the solid phase of snow exceeds soil data by almost 11 times, followed by lead at 6.3 times, chromium and copper exceed 3.3 times and other metals (iron, nickel, zinc and arsenic) vary between 1.3 and 1.8 times. The exception is manganese, whose content in the soil is more than 37 times higher than that of this metal in the snow.

The mass concentration of the heavy metals/ metalloid in question is lowest in vegetables. The highest values were found for iron, which ranged from 0.4 to 1.1 mg/kg. Despite the extremely high concentration of manganese in the soil—22,125 mg/kg—its content in potatoes decreased by a factor of 1865 and amounted to 11.86 mg/kg (range 7–31 mg/kg). Zinc (15–25 mg/kg), copper (2.7–20 mg/kg), nickel (0.5–3.6 mg/kg), chromium (0.4–1.1 mg/kg) and lead (0.07–0.37 mg/kg) followed in decreasing order.

Comparing the data on the mass concentration of heavy metals/ metalloid in tomatoes and potatoes, the following conclusions can be drawn. The series of decreasing concentrations is identical, but there are variations in the data. For example, the levels of iron and copper in potatoes are on average 1.5 times higher than in tomatoes. All the other metals have similar values in the two vegetables, with a slight bias towards potatoes. The variation in the mass concentration of microelements in tomatoes was as follows: Cr (0.5–1.7 mg/kg), Mn (9–18 mg/kg), Fe (25–75 mg/kg), Co (0.02–0.16 mg/kg), Ni (0.4–2.2 mg/kg), Cu (3.6–9.6 mg/kg), Zn (11–32 mg/kg), Pb (0.06–0.12 mg/kg), As and Cd—less than 0.1 mg/kg.

Compared to other studies [43] of snow cover in Kazakhstan that were done in a small settlement in the East Kazakhstan region, our study found that the amount of cobalt, nickel and copper in the snow cover of gardens in the city of Pavlodar and nearby settlements is almost the same, though there is a higher concentration of most metals. For example, the level of chromium in the Pavlodar snow is 6.2 times higher than in North Kazakhstan’s settlement, but the levels of zinc and cadmium in our study are 1.2 and 1.7 times lower, respectively. In studies of the East Kazakhstan region, snow samples were taken in areas directly influenced by anthropogenic factors such as vehicles, stove heating and boiler houses. Thus, the anthropogenic influence of the urban environment on snow cover is high in dachas and vegetable gardens.

The results of studies of soil cover on agricultural land in the Kostanay region of the Republic of Kazakhstan [44] show low levels of heavy metal content compared to Pavlodar vegetable gardens. Thus, in Pavlodar, the content of lead in soils exceeds the content in agricultural lands of Kostanay by 3.4 times, followed by arsenic by 3 times, etc., except for cadmium, the concentration of which, on the contrary, is 2 times lower in our studies.

Compared to the urban soils of the Republic of Kazakhstan, which are the most contaminated with heavy metals (Balkhash, Ust-Kamenogorsk, Ridder and Shymkent), the average metal concentrations varied between 251 and 442 mg/kg for Pb, 5–9 mg/kg for Cd, 8–138 mg/kg for Cu, 87–178 mg/kg for Zn and 2–5 mg/kg for Cr (Ramazanova et al., 2021) [45].The soils of vegetable gardens and dachas in Pavlodar and its surroundings contain large amounts of chromium and zinc, which exceed the average and maximum values of the data presented by Ramazanova et al., 2021 [45]. For cadmium and lead, the values in our study are significantly lower than in the above studies of urban soils; the remaining metals are within the range of values.

### 3.2. Assessment of Heavy Metal Contamination

An indicator of heavy metal contamination of snow (solid fraction) and soil is the contamination coefficient relative to the background concentration.

**Figure 2:**
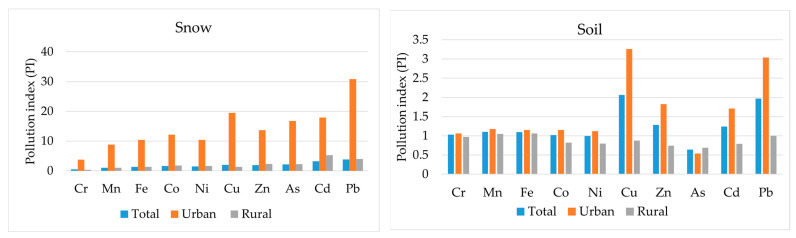
Indices of heavy metals and metalloid pollution of snow and soil covers.

Figure 2 shows the contamination coefficients of different media. The highest values of the snow contamination coefficient for all metals are found in the city’s vegetable gardens. The descending series of average contamination index values in the snow cover of the city gardens is as follows: Pb > Cu > Cd > As > Zn > Co > Ni > Fe > Mn > Cr. The minimum exceedance of the background indicators relates to chromium (3.75), which corresponds to severe pollution according to the pollution classification of this coefficient. Thus, all of the other metals in urban snow are classified as highly polluted. For village gardens, the pollution index varies from 0.3 for chromium to 5.3 for cadmium. The level of contamination of snow is low or medium for most metals and high for cadmium and lead.

There is not such a big difference between urban and rural areas for heavy metal soil pollution, although there is still a tendency for urban areas to exceed these values. The highest pollution coefficients are Cu > Pb > Zn > Cd > Mn > Co > Fe > Ni > Cr > As. In the soils of urban gardens, arsenic has a very low coefficient, indicating an uncontaminated area; the maximum values are recorded for copper (3.3) and lead (3.04), corresponding to severe contamination. The remaining metals range between 1 and 2, indicating low levels of contamination. For most metals, rural soils are uncontaminated compared to background levels, with only the values for iron, magnesium and lead indicating low levels of contamination.

**Figure 3:**
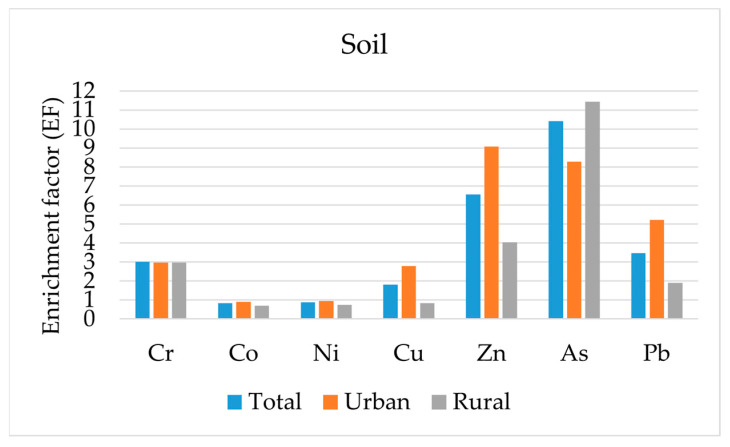
Enrichment factor of soils in Pavlodar with heavy metals/ metalloid.

The enrichment factor (EF) allows us to estimate the degree of intensity of anthropogenic activity relative to the clark concentration (geochemical background). In our study, the concentration of iron (Fe) was used as the reference background concentration. As a result of the calculations, data on soil accumulation in different vegetable growing areas were obtained (Figure 3).

In general, the enrichment of soils with heavy metals/metalloid in the city is represented in the following series: Mn > As > Zn > Pb > Cr > Cu > Ni > Co > Cd. With maximum values characteristic of manganese (50.5), arsenic (10.4), zinc (6.6), lead (3.5) and chromium (3.03). Nickel, cobalt and cadmium have values below 1, which corresponds to a normal distribution in relation to concentrations in the earth’s crust.

Maximum allowable concentrations (MACs) of pollutants are used to regulate the content of heavy metals in soils in the Republic of Kazakhstan. In order to determine the degree of compliance with these soil standards, in our study we determined the degree of exceedance of the maximum allowable concentrations of heavy metals in the soils of urban and rural gardens. We used the MPCs according to Hygienic standards for the safety of the environment, 2021 [39], for lead and arsenic, the WHO standard [40] for chromium and the Russian GOST standards (Hygienic Standards HS 2.1.7.2041-06) [38] for other metals (Table 2).

Most metals do not exceed established standards for soil content. However, magnesium exceeds the MPC by 28.6–35.7 times, an excess indicating extreme pollution. Zinc also exceeds standards in a wide range, from 1.6 to 10.9 times the MPC in all study areas. The arsenic content in urban gardens reaches 6, while the minimum value at point 6 is 0.05. The arsenic content is higher in villages than in cities, which distinguishes this metal’s behavior from others. The concentrations of cobalt and nickel are higher than the standard values in urban gardens, while in rural gardens, the concentrations of these metals are normal. The maximum exceedance values of the MPCs for chromium, copper and lead also apply to urban gardens, and the exceedance is 10.4, 3.9 and 2.25, respectively. Thus, manganese and zinc are the main pollutants in both urban and rural areas. Only cadmium has minimum values with respect to the maximum permissible concentration, whereas the maximum values of the other metals exceed the city’s maximum concentrations.

The hygienic standards for controlling the concentration of heavy metals in food have been adopted as the acceptable level of contamination (mg/kg) for different products in the Republic of Kazakhstan (Sanitary and epidemiological requirements for food products, 2010) [41]. This legislation is no longer in force and standards for heavy metals in vegetables have had to be obtained from other sources. Thus, the limiting indicators of heavy metal pollution in vegetables adopted by WHO [41] for chromium, nickel, copper and lead, literature sources [19]. for cobalt and sanitary rules and regulations of the USSR for zinc (SanPiN 42-123-4089-86) [42] were taken into account (Table 3). 

Of the metals considered, only zinc exceeds the MPC for both potatoes and tomatoes. In some areas, the copper content also exceeds the standards, although the average value is within the normal range. In general, chromium and cobalt are characterized by an excess of the standard concentration for tomatoes, and other metals accumulate more in potatoes.

**Figure 4:**
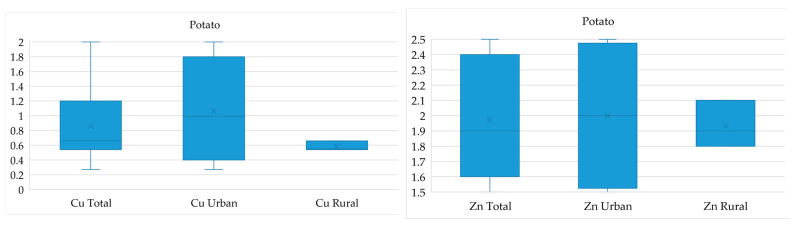

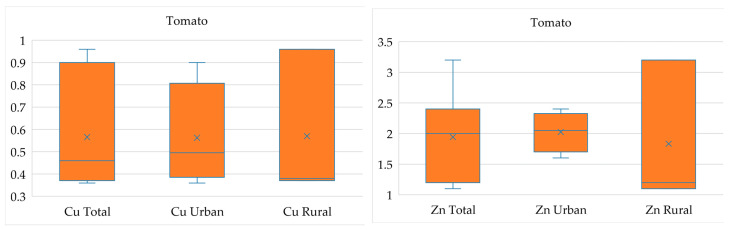
Exceedance of soil MPC indicators by average values of heavy metal content in vegetables in the city of Pavlodar.

Since the highest excesses were found for zinc and copper, Figure 4 shows the spatial patterns of the content of these metals in vegetables in rural and urban gardens, as well as in the city as a whole.

It was found that the concentration of zinc and copper in the city largely exceeds the MPC, in contrast to rural gardens, where the range varies from 1.8 to 2.1 for zinc and from 0.54 to 0.66 for copper. The opposite situation was observed for tomatoes, where the high content of the metals studied corresponds to village gardens.

**Figure 5:**
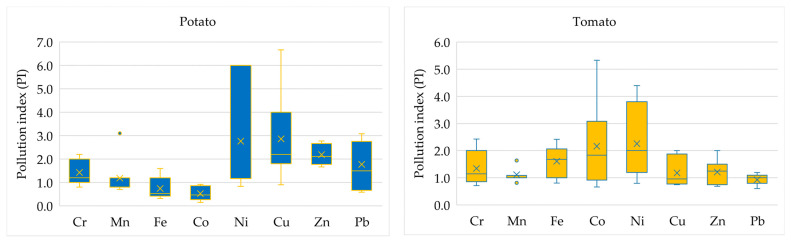
Pollution index of metal and metalloid content in vegetables.

The pollution index relative to the background for potatoes and tomatoes is shown in Figure 5.

Relative to the background, nickel is the most important contaminant for the vegetables studied, with an excess of 6 (very high contamination) for potatoes and 4.4 (high contamination) for tomatoes. The average value of soil contamination with this metal refers to the average level of contamination. For tomatoes, cobalt is also a strong contaminant; its value is 2.2 times higher than the background value, and the maximum value is 5.3. The remaining metals are at a low level of contamination. In potatoes, lead, zinc, copper and nickel are classified as moderately polluted, and the metals are divided into non-polluted and moderately polluted.

### 3.3. Bioaccumulation of Heavy Metals in Vegetables

**Figure 6:**
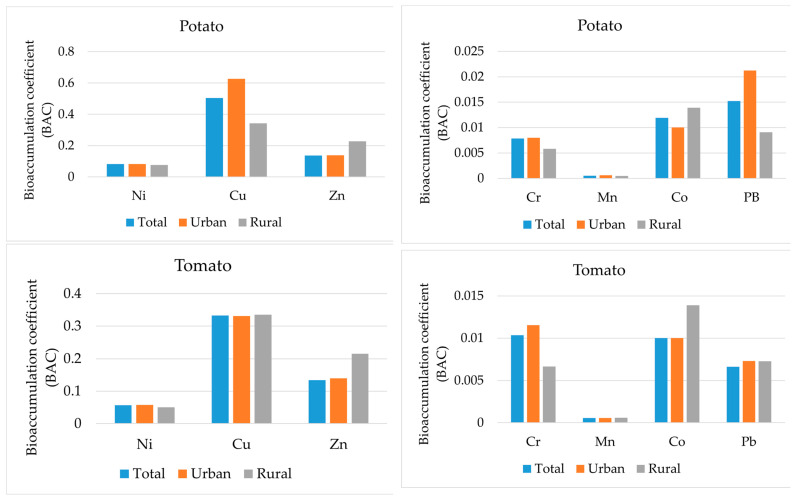
Bioaccumulation coefficient of vegetables.

Considering the bioaccumulation coefficient for vegetables grown in the study areas, the following results were obtained (Figure 6). The majority of metals in both potatoes and tomatoes have very low accumulation levels. This means that despite high concentrations of contaminants in the soil, plants have mechanisms to prevent the metals from entering. In the case of potatoes, copper is moderately to highly bioaccumulative, ranging from 0.3 to 0.6, and zinc is at negligible levels (0.1–0.2). The same picture emerges for tomato, where copper has a moderate transfer coefficient of 0.3 and zinc varies between 0.1 and 0.2, also belonging to the insignificant transfers.

As regards the distribution between urban and rural gardens, the bioaccumulation coefficient varies slightly. For potatoes, the highest correlation with the distribution of heavy metals in the soil is observed. The predominance of copper and lead in urban soils is also present in potato samples from urban areas. The planting location’s effect on the transfer of pollutants from soils to tomatoes is insignificant. Thus, we observe less constant accumulation of pollutants from soils for above-ground vegetables and more for root vegetables. This may be a consequence of the high mobility of these metals naturally occurring in soils, as well as their lower retention in soils compared to other toxic elements [46].

### 3.4. Health Risk Assessment

For both local adults and children, the human health risk of exposure to trace elements through consumption of contaminated local crops was assessed.

To calculate the carcinogenic and non-carcinogenic risks, the daily intake of vegetables for adults and children was calculated and presented in Table 4. In general, according to the maximum tolerable intakes (MDIs) presented in different sources [47,48], the daily consumption of all heavy metals does not exceed the standards. However, potatoes have the highest values for both adults and children, as their average consumption is higher than that of tomatoes.

Analysis of the non-carcinogenic risk of exposure to heavy metals revealed that no single metal posed a significant threat to children and adults, as a hazard coefficient value less than one indicates no consumer risk. Thus, in our studies, the carcinogenic risk of the heavy metals in question is low for children and adults. Potatoes have slightly higher levels than tomatoes. Lead has the lowest levels, so it has virtually no impact on human health, with slightly higher levels of nickel and chromium in both vegetables for children.

The Hazard Index (HI) and the Carcinogenic Risk (CR) were considered as indicators of the potential risk to human health from exposure to several potentially toxic elements (Table 5 and Table 6). If the hazard index value is less than 1, adverse health effects are unlikely. In general, the non-carcinogenic risk for potatoes and tomatoes is low. Spatially, the hazard index for consuming potatoes in cities is 1.3 higher for both adults and children than in rural gardens. Eating potatoes also has a low carcinogenic risk. Values above 10^−4^ represent significant health effects and risks, while values below 10^−6^ are unlikely to cause health effects. The carcinogenic risk of consuming potatoes in an urban area is 1.6 times higher for adults and children than in the countryside, according to the hazard coefficient.

The risk of adverse effects from urban and rural consumption of tomatoes is lower than for potatoes, but this is also associated with lower tomato consumption. However, the overall non-carcinogenic and carcinogenic risks of tomatoes do not differ much between urban and rural gardens and are almost identical, indicating that tomatoes have a high selectivity.

### 3.5. Sources of Pollution

The results show the impact of industry and transport on plants and, consequently, on human health when consuming vegetables. Although the average health risk is not high, metals such as chromium, lead, nickel, cadmium and zinc are of concern as the maximum permissible concentrations of these metals exceed the standards.

Another aim of the study is to try to identify the sources of contamination of vegetables in the territory of the city of Pavlodar as well as in the nearest settlements. The dacha plots of site 1 are situated in the southern direction of the Northern Industrial Zone, approximately 1.5 km away from the largest industrial facility, the Pavlodar Oil Refinery. Meanwhile, sites 2 and 4 of the city’s vegetable gardens were directly impacted by motor transport, including personal vehicles, near the vegetable planting areas. The southern part of the city (sites 6, 7) was also affected by the Eastern Industrial Zone with aluminum production, CHPP-1, ash dumps and sludge pits.

In order to identify the sources of pollution by heavy metals and metalloids, the emissions of the main industrial enterprises in the city of Pavlodar were analysed according to the results of the industrial environmental control (Table S1). The amounts of heavy metals/metalloids emitted in pure form are not high. However, the ash and dust emissions of the industrial enterprises in Pavlodar contain the following compositions of elements: lead—2.86 mg/kg; cadmium—0.23 mg/kg; mercury—6.59 mg/kg; arsenic—0.28 mg/kg; fluorine—10.0 mg/kg; antimony—0.37 mg/kg; beryllium—0.017 mg/kg; selenium—0.53 mg/kg; tellurium—0.1 mg/kg [49]. The main sources of dust and ash emissions are CHPP-2, CHPP-3, followed by KSP “Steel” (metallurgical production). Other emissions are as follows: aluminum—78.877 t/year; petrol—48.74 t/year, emitted by the Pavlodar Petrochemical Plant; manganese—18 t/year; iron—16.915 t/year; emitted by KSP “Steel”; as well as 1.45 t/year of iron emitted by “Kasting” LLP (metallurgical production). The largest amounts of chromium (6 t/year), zinc (0.3135 t/year) and wood dust (6.741 t/year) are emitted by the pipe mill. JSC “Kaustik” (chemical plant) emits 0.175 tonnes/year of chlorine, 0.313 tonnes/year of zinc [50]. The analysis showed that the maximum emissions from aluminum production are from aluminum: 133.5 t/year from the aluminum smelter and 433.6 t/year from the electrolysis plant [51]. It should be noted that, according to the results of industrial environmental control, none of the industrial facilities exceeds the normative indicator of pollutant emissions adopted in the Republic of Kazakhstan.

It is important to identify the sources of pollution that influence the accumulation of pollutants in vegetables. For instance, Weissmannová’s 2019 [30] studies on the relationship between pollution sources, heavy metals and metaloids reveal a connection between pollutants and industrial and traffic emissions. Studies confirm that most metals (Pb, Cu, Cd) are grouped by NSA analysis and originate only from anthropogenic sources (metallurgy, coal burning, transport). The high iron and manganese content may also be due to weathering of the parent rock and emissions from iron production, rolling mills, blast furnaces and so on. Emissions of the Fe, Cr and Cd groups of metals and the Pb-Cu group are due to coal combustion in industry and local water heating. In the city of Pavlodar, this refers to the operation of CHPP-1, 2 and 3, as well as to the areas of urban and village vegetable gardens. Vegetable gardens are usually adjacent to houses with stove heating, and snow accumulates all emissions during the winter period (from November to April). High concentrations of lead in all environments and in vegetables are caused by coal burning, metallurgy and industrial waste. Zinc is emitted in the production of stainless steel (steel production). Zinc, lead and copper are also widely emitted by car exhaust [33]. In 2022, emissions of pollutants from mobile sources in the Pavlodar region will amount to 43 thousand tonnes [20].

Previous studies [52] also support the assumption that motor vehicle emissions and the deposition of chemical elements in atmospheric dust are the primary sources of heavy metal/metalloid contamination of vegetables, based on the reviewed data. Domestic coal combustion can be considered potentially problematic, with serious environmental and health consequences, as can motor vehicle emissions. We recommend using high-quality coal and petrol for domestic use, as well as protecting plants from car exhaust. The data obtained on heavy metal/metalloid accumulation in vegetables and comparison with concentrations in snow and soil can be used for further research to identify patterns and use only one medium to determine contaminant levels in the snow-soil-plant system. Thus, methods already exist to assess spatial air pollution based on snow cover [27]. In addition, the results of this study may motivate ecologists, managers and health workers to educate the public about the risks of consuming vegetables grown on contaminated soils, which may help reduce health hazards. In addition, it is recommended for managers to provide publicly available information on the standards of permissible level of heavy metal contamination of food products, including fruits and vegetables. The authors recommend regular monitoring of heavy metal and metalloid levels in snow, soil and vegetables to prevent significant metal accumulation in the food chain and mitigate public health risks.

## 4. Conclusions

The study showed that the highest concentrations of trace elements are found in the solid phase of snow and they decrease in the following order: Fe (26,000) > Mn (592.5) > Cr (371.3) > Zn (338.8) > Pb (161.9) > Cu (142.5) > Ni (30.9) > As (15.1) > Co (12.1) > Cd (2.6). Pollutant concentrations in the liquid phase of snow (meltwater) were lower than in solid snow sediments and soils, but higher than in vegetables.

In the soils of Pavlodar, where vegetables are grown, the average concentrations of heavy metals/ metalloid decrease in the following order: Mn (22,125) > Fe (20,375) > Zn (246.9) > Cr (109.5) > Cu (39.3) > Pb (25.6) > Ni (22.4) > As (9) > Co (6.6) > Cd (0.2). The comparison revealed that the metal content in snow significantly exceeds its concentration in soil.

In urban gardens, the snow pollution coefficient was highest, with a decreasing series of pollution indices: Pb > Cu > Cd > As > Zn > Co > Ni > Fe > Mn > Cr. The minimum background value exceedance for Cr (3.75) indicates severe pollution. The remaining metals in the urban snow indicate a very high level of pollution. In the village gardens, the pollution index ranged from 0.3 (Cr) to 5.3 (Cd), with low to moderate pollution for most metals but high pollution for Cd and Pb.

Most metals in the soil do not exceed established standards, with the exception of magnesium, which exceeds the MPC by 28.6–35.7 times, corresponding to extreme contamination. Zinc also exceeds the standards by 1.6–10.9 times in all areas.

For vegetables, only zinc exceeds the MPC for potatoes and tomatoes. In some places, copper exceeds the standards, but the average values are within the normal range. Nickel is the most important pollutant for the vegetables studied, exceeding the MPC by a factor of 6 in potatoes (very high contamination) and by a factor of 4.4 in tomatoes (high contamination). Cobalt is also a strong contaminant for tomatoes, exceeding background levels by a factor of 2.2, with a maximum value of 5.3.

The bioaccumulation factor for vegetables shows that most metals in potatoes and tomatoes are at very low accumulation levels. For potatoes, copper ranges from moderately to highly bioaccumulative (0.3–0.6) and zinc to negligible levels (0.1–0.2). For tomatoes, the picture is similar: copper exhibits a coefficient of 0.3, while zinc fluctuates between 0.1 and 0.2.

In general, the non-carcinogenic risk of potatoes and tomatoes is low. For adults and children, the hazard index for consuming potatoes in the city is 1.3 times higher than in rural gardens. The carcinogenic risk of eating potatoes is also low, but it is 1.6 times higher in the city than in the countryside.

## Figures and Tables

**Table 1:** Average content of heavy metals/metalloid in different environments.

	Cr	Mn	Fe	Co	Ni	Cu	Zn	As	Cd	Pb
Snow SPS (mg/kg)	371.6 ± 178.8	592.5 ± 52	26,000 ± 4690.4	12.06 ± 5.3	30.9 ± 12	142.5 ± 126.3	338.8 ± 203.4	15.1 ± 10.6	2.6 ± 1.73	161.9 ± 91.2
Snow LSS (μg/L)	1.7 ± 1.4	29.1 ± 18.6	222.8 ± 315.8	less than 0.1	less than 0.1	3.5 ± 2.6	28.3 ± 15.3	2.9 ± 0.9	less than 0.1	5.5 ± 2.41
Soils (mg/kg)	109.5 ± 17.9	22,125 ± 1959.4	20,375 ± 1635	6.6 ± 1.8	22.4 ± 5.7	39.2 ± 40.1	246.9 ± 162	9 ± 3.4	0.2 ± 0.14	25.6 ± 23.3
MPC in soil (mg/kg)	100 ***	700 *	-	5 *	20 *	33 *	55 *	2 **	0.5 *	32 **
Potato (mg/kg)	0.7 ± 0.3	11.9 ± 8.6	74.3 ± 47.3	0.07 ± 0.04	1.7 ± 1.4	8.6 ± 5.8	19.7 ± 3.8	less than 0.1	0.05 ± less than 0.1	0.2 ± 0.1
Tomato (mg/kg)	0.94 ± 0.44	12.29 ± 2.75	49.86 ± 18.32	0.07 ± 0.05	1.13 ± 0.69	5.66 ± 2.57	19.43 ± 7.3	less than 0.1	0.08 ± less than 0.1	0.09 ± 0.02
MPC in plants (mg/kg)	1.3 *	-	-	0.2 ***	10 *	10 *	10 **	-	-	2 *

MPC—maximum permissible concentration. MPC in soil: * Russian GOST standards (Hygienic Standards HS 2.1.7.2041-06) [38]; ** Hygienic standards for the safety of the environment, 2021 [39]; *** WHO standard (Alengebawy et al., 2021) [40]. MPC in plants: * WHO (JECFA, 2003) [41]; ** SanPiN 42-123-4089-86 [42]; *** Geldymamedova, 2007) [19].

**Table 2:** Exceedance of MPC indicators of soil by average values of heavy metals/metalloid content in soil in the city of Pavlodar.

Element	MPC Exceeded in Urban Gardens	MPC Exceeded in Village Gardens
Cr	0.91–1.4	0.85–1.2
Mn	31.43–35.71	28.57–32.86
Co	1.2–1.8	0.6–1.4
Ni	1–1.65	0.65–1.1
Cu	0.52–3.94	0.48–0.52
Zn	2.64–10.18	1.55–3.36
As	0.05–6	2.2–5.5
Cd	0.28–0.92	0.22–0.36
Pb	0.44–2.25	0.22–0.66

**Table 3:** Exceedance of soil MPC indicators by average values of heavy metal content in vegetables in the city of Pavlodar.

Microelement	Cr		Co		Ni		Cu		Zn		Pb	
Vegetable	Potato	Tomato	Potato	Tomato	Potato	Tomato	Potato	Tomato	Potato	Tomato	Potato	Tomato
Range	0.31–0.85	0.38–1.31	0.02–0.12	0.1–0.8	0.05–0.36	0.04–0.22	0.27–2	0.36–0.96	1.5–2.5	1.1–3.2	0.04–0.19	0.03–0.06
Average	0.55	0.73	0.07	0.33	0.17	0.11	0.86	0.57	1.97	1.94	0.11	0.05

**Table 4:** Critical daily intake (CDI) of potentially toxic metals (mg/kg-day).

CDI	Cr	Mn	Fe	Ni	Cu	Zn	Pb
ADULT Potato	1.2 × 10^6^	1.99785 × 10^5^	1.25 × 10^4^	2.79 × 10^6^	1.45 × 10^5^	3.32 × 10^5^	3.59 × 10^7^
CHILD Potato	2.11 × 10^6^	3.49623 × 10^5^	2.19 × 10^4^	4.89 × 10^6^	2.53 × 10^5^	5.81 × 10^5^	6.28 × 10^7^
ADULT Tomato	2.84 × 10^7^	3.70254 × 10^6^	1.5 × 10^5^	3.4 × 10^7^	1.7 × 10^6^	5.86 × 10^6^	2.8 × 10^8^
CHILD Tomato	4.75 × 10^7^	6.47945 × 10^6^	2.63 × 10^5^	5.95 × 10^7^	2.98 × 10^6^	1.02 × 10^5^	4.9 × 10^8^

**Table 5:** Total non-carcinogenic (HI) and carcinogenic (LCR) risks of potato consumption for adults and children.

HI				LCR			
Potato							
Urban		Rural		Urban		Rural	
Adults	Children	Adults	Children	Adults	Children	Adults	Children
1.16412 × 10^3^	2.03721 × 10^3^	8.66988 × 10^4^	1.517229 × 10^3^	3.145 × 10^6^	5.5037 × 10^6^	1.9805 × 10^6^	3.45497 × 10^6^

**Table 6:** Total non-cancer (HI) and cancer (LCR) risks of tomato consumption for adults and children.

HI				LCR			
Tomato							
Urban		Rural		Urban		Rural	
Adults	Children	Adults	Children	Adults	Children	Adults	Children
1.36554 × 10^4^	2.32904 × 10^4^	1.33493 × 10^4^	2.27811 × 10^4^	4.7386 × 10^7^	8.2926 × 10^7^	4.2242 × 10^7^	7.3924 × 10^7^

## Data Availability

The authors used data published by the National Hydrometeorological Service of the Republic of Kazakhstan. Monthly newsletter on the state of the environment (https://www.kazhydromet.kz/ru/ecology/ezhemesyachnyy-informacionnyy-byulleten-o-sostoyanii-okruzhayuschey-sredy, accessed on 22 March 2024).

## Supplementary Materials

The following supporting information can be downloaded at: https://www.mdpi.com/article/10.3390/ijerph21081002/s1, Table S1: Emissions of some pollutants from industrial enterprises in the city of Pavlodar (kg/year) (source: industrial environmental control reports).

## References

[1] Hu Y., Liu X., Bai J., Shih K., Zeng E., Cheng H. (2013). Assessing heavy metal pollution in the surface soils of a region that had undergone three decades of intense industrialization and urbanization. Environ. Sci. Pol. Res..

[2] Zhang F., Meng B., Gao S., Hough R., Hu P., Zhang Z., Yu S., Li K., Liu Z., Cui S. (2021). Levels, Inventory, and Risk Assessment of Heavy Metals in Wetland Ecosystem, Northeast China: Implications for Snow Cover Monitoring. Water.

[3] Szwed M., Kozłowski R. (2022). Snow cover as an indicator of dust pollution in the area of exploitation of rock materials in the świętokrzyskie mountains. Atmosphere.

[4] Liu Z., Zhang Q., Han T., Ding Y., Sun J., Wang F. (2015). Heavy metal pollution in a soil-rice system in the Yangtze river region of China. Int. J. Environ. Res. Public Health.

[5] Danila V., Vasarevičius S. Theoretical evaluation of heavy metals migration and sorption in soil. Proceedings of the “Environmental Engineering” 10th International Conference.

[6] Luo Y., Wang Z., Zheng C. (2022). Simulation of heavy metals migration in soil of rare earth mining area. J. Environ. Inf. Let..

[7] Vallero D.A. Persistent, Bioaccumulative, and Toxic Pollutants. AccessScience 2023. https://www.accessscience.com/content/article/a500920.

[8] Csuros M., Csuros C. (2002). Environmental Sampling and Analysis for Metals.

[9] (1992). Methodological Guideline on Determination of Heavy Metals in Agricultural Soils and Crops.

[10] Witkowska D., Słowik J., Chilicka K. (2021). Heavy Metals and Human Health: Possible Exposure Pathways and the Competition for Protein Binding Sites. Molecules.

[11] Monib A.W., Niazi P., Azizi A., Sediqi S., Baseer A.Q. (2024). Heavy Metal Contamination in Urban Soils: Health Impacts on Humans and Plants: A Review. Eur. J. Theor. App. Sci..

[12] Ebrahimi M., Razi S., Keshavarz-Fathi M., Khalili N., Rezaei N. (2020). Effects of lead and cadmium on the immune system and cancer progression. J. Environ. Health Sci. Eng..

[13] Zhang W., Ma L., Abuduwaili J., Ge Y., Issanova G., Saparov G. (2019). Distribution characteristics and assessment of heavy metals in the surface water of the syr darya river, kazakhstan. Pol. J. Environ. Stud..

[14] Kim B., Poulsen M., Margulies J., Dix K., Palmer A., Nachman K. (2014). Urban community gardeners’ knowledge and perceptions of soil contaminant risks. PLoS ONE.

[15] Mitchell R., Spliethoff H., Ribaudo L., Lopp D., Shayler H., Marquez-Bravo L., McBride M. (2014). Lead (pb) and other metals in new york city community garden soils: Factors influencing contaminant distributions. Environ. Pol..

[16] Antisari L., Orsini F., Marchetti L., Vianello G., Gianquinto G. (2015). Heavy metal accumulation in vegetables grown in urban gardens. Agron. Sust. Dev..

[17] Cooper A., Felix D., Alcantara F., Zaslavsky I., Work A., Watson P., Schroeder J. (2020). Monitoring and mitigation of toxic heavy metals and arsenic accumulation in food crops: A case study of an urban community garden. Plant Direct.

[18] Montaño-Lopez F., Biswas A. (2021). Are heavy metals in urban garden soils linked to vulnerable populations? a case study from Guelph, Canada. Sci. Rep..

[19] Geldymamedova E.A. (2007). Heavy Metals in Soils of Pavlodar, Republic of Kazakhstan. Ph.D. Thesis.

[20] (2022). Monthly Newsletter on the State of the Environment. kazhydromet.kz. https://www.kazhydromet.kz/ru/ecology/ezhemesyachnyy-informacionnyy-byulleten-o-sostoyanii-okruzhayuschey-sredy.

[21] Koroleva E.A., Rakhimbek S.K., Tupov S.S. (2019). Medical and geographical aspects of monitoring of population morbidity. Hyg. Sanit..

[22] Imasheva B.S., Askarov K.A., Imashev M.S., Kereybaeva A.N., Tokbergenov E.T., Kuatbaeva A.M. (2022). Assessment of the health risk of the population living in the region of the location of the facilities of the Pavlodar aluminum plant of JSC “Aluminum of Kazakhstan”. Nauka I Zdr. [Sci. Healthc.].

[23] Meteoizdat M. (1982). Methodological Recommendations for Conducting Field and Laboratory Studies of Soils in Monitoring Environmental Pollution by Metals.

[24] Baisholanova S.S. (2017). Agroclimatic Resources of the Pavlodar Region: Scientific and Applied Reference Book.

[25] (1992). Guidelines for Determination of Heavy Metals in Soils of Agricultural Land and Plant Products.

[26] Unified Methods of Natural Environmental Background Monitoring (LAM Rosgidromet, RAN, Moscow, 1986).

[27] (1990). Methodological Recommendations for Assessing the Degree of Atmospheric Air Pollution in Populated Areas by Metals Based on Their Content in Snow Cover and Soil.

[28] (2016). Methods of Quantitative Chemical Analysis.

[29] (2019). Water Quality—Application of Inductively Coupled Plasma Mass Spectrometry (ICP-MS).

[30] (2008). M-MVI-2008 Guidelines; Method of Measurement Mass Fractions of Elements in Samples of Soils, Bottoms and Bottom Sediments Bottom Sediments by Atomic Emission and Atomic Emission and Atomic Absorption Spectrometry.

[31] (2008). Protection of Nature. Soils. Classification of Chemicals for Pollution Control.

[32] Weissmannová H.D., Mihočová S., Chovanec P., Pavlovský J. (2019). Potential ecological risk and human health risk assessment of heavy metal pollution in industrial affected soils by coal mining and metallurgy in ostrava, Czech Republic. Int. J. Environ. Res. Public Health.

[33] Sutherland R.A. (2000). Bed sediment-associated trace metals in an urban stream, Oahu, Hawaii. Environ. Geol..

[34] Vinogradov A.P. (1962). Average content of chemical elements in the main types of igneous rocks of the Earth’s crust. Geochemistry.

[35] USEPA (2001). Baseline Human Health Risk Assessmen.

[36] Manea D.N., Ienciu A.A., Stefan R., Shmuleac I.L., Gergen I.I., Nica D.V. (2020). Health Risk Assessment of Dietary Heavy Metals Intake from Fruits and Vegetables Grown in Selected Old Mining Areas—A Case Study: The Banat Area of Southern Carpathians. Int. J. Environ. Res. Public Health.

[37] Lu X., Zhang X., Li L.Y., Chen H. (2014). Assessment of metals pollution and health risk in dust from nursery schools in Xi’an, China. Environ. Res..

[38] (2006). Maximum permissible concentrations (MPC) of chemicals in soil.

[39] (2021). Hygienic standards for the safety of the environment. Order of the Minister of Health of the Republic of Kazakhstan, No. 22595.

[40] Alengebawy A., Abdelkhalek S.T., Qureshi S.R., Wang M.-Q. (2021). Heavy Metals and Pesticides Toxicity in Agricultural Soil and Plants: Ecological Risks and Human Health Implications. Toxics.

[41] JECFA (2003). Summary and Conclusions of the 61st Meeting of the Joint FAO/WHO.

[42] (1986). Maximum Permissible Concentrations of Heavy Metals and Arsenic in Food Raw Materials and Food Products.

[43] Temirzhanova L.A., Dyusembaeva E., Madina T., Lukashenko S., Yazikov E.G., Shakenov E.Z. (2020). Elemental composition of snow cover solid phase in small settlements (the case of Dolon Village, Republic of Kazakhstan). Bull. Tomsk Polytech. Univ. Geo Assets Eng..

[44] Zhyrgalova A., Yelemessov S., Ablaikhan B., Aitkhozhayeva G., Zhildikbayeva A. (2024). Assessment of potential ecological risk of heavy metal contamination of agricultural soils in Kazakhstan. Braz. J. Biol..

[45] Ramazanova E., Lee S.H., Lee W. (2021). Stochastic risk assessment of urban soils contaminated by heavy metals in Kazakhstan. Sci. Total Environ..

[46] Orisakwe O.E., Dagur E.A., Udowelle N.A. (2017). Lead levels in vegetables from Artisanal Minning sites of Dilimi River, Bukuru and Barkin Ladi North Central Nigeria: Cancer and Non-Cancer Risk Assessment. Asian Pac. J. Cancer Prev..

[47] Arora M., Kiran B., Rani S., Rani A., Kaur B., Mittal N. (2008). Heavy metal accumulation in vegetables irrigated with water from different sources. Food Chem..

[48] Kormoker T., Proshad R., Islam M., Tusher T., Uddin M., Khadka S., Chandra K., Sayeed A. (2020). Presence of toxic metals in rice with human health hazards in Tangail district of Bangladesh. Int. J. Environ. Health Res..

[49] Azhaev A.A. (2007). Assessment of the Ecological State of Pavlodar According to the Geochemical Study of Liquid and Dust Atmospheric Precipitation. Ph.D. Thesis.

[50] (2022). Committee for Technical Regulation and Metrology of the Ministry of Industry and Trade of the Republic of Kazakhstan: Astana, Kazakhstan. https://www.gov.kz/memleket/entities/mti-ktrm?lang=en.

[51] Faurat A.A., Azhaev G.S., Kakezhanova S.K., Dossova M.T. (2024). Heavy Metals Contamination in Snow Cover of Pavlodar (Kazakhstan). Bulletin of the Karaganda University. Biol. Med. Geo. Ser..

[52] Azhayev G., Esimova D., Sonko S.M., Safarov R., Shomanova Z., Sambou A. (2020). Geoecological Environmental Evaluation of Pavlodar Region of The Republic of Kazakhstan as a Factor of Perspectives for Touristic Activity. GeoJ. Tour. Geosit..

