# Supplementary material for: Assessment of Heavy Metal Contamination and Health Risks in “Snow Cover–Soil Cover–Vegetation System” of Urban and Rural Gardens of an Industrial City in Kazakhstan
Source: Int J Environ Res Public Health. 2024 Jul 30;21(8):1002. doi: 10.3390/ijerph21081002 (PMC11353635; doi:10.3390/ijerph21081002)
Supplement: Supplementary file 1 [file ijerph-21-01002-s001.zip › ijerph-3046805-supplementary.pdf]

Supplementary material

Table S1. Emissions of some pollutants from industrial enterprises in the city of Pavlodar (kg/year) (source: industrial environmental control reports)

Name of pollutant	Emissions of pollutants from industrial enterprises Northern industrial zone of Pavlodar (kg/year) (Faurat et al, 2024) [48]						Emissions of pollutants from industrial enterprises in the Eastern industrial zone of Pavlodar (kg/year) (Faurat et al, 2023) [49]	
	Fuel and energy complex			Metallurgical industry		Chemical industry	Aluminum industry	
	PNHZ-petrochemical production	CHPP-2	CHPP-3	KSP "Steel" - pipe rolling production	LLP "Casting" Steel Plant	JSC "Caustic"	JSC "Aluminum of Kazakhstan", CHPP - 1	JSC "Kazakhstan Electrolysis Plant"
Iron	1.1	5.1539	2176.3	16915.4	1450.8	265.0012	9.255	0.101138
Manganese and its compounds	0.051	11,453	50,601	18000.84	5,289	5.555	0.23	0.0036362
Aluminum	78,877.7			1322.7		0.0037	133,564	433.591175
Chromium	0.0446	0.3279	1.46	6.0	0.04	0.0253	0.0241	0.0000012
Chlorine						175.166		0.1436759
Nickel oxide	0.005	0.203	0.0025	0.0003	0.0039	0.0029	0.0002774	
Lead		0.0175		0.002		0.0004	0.000022	
Copper		1.712	20,915	0.052		0	0.0166	
Zinc		0.0292		313.5		0	0.0000038	
Petrol	48,740.92		50.77	35.2		675.18	0.632	0.0113740
Kerosene		5.63		14.2	81.5	0	1.657	
Inorganic dust	188 492	1,177,692	4 885 213	304,247.2	220,000	1436.5539 (+ suspended solids)	37457.9823 (particulate matter)	806.296
Abrasive dust	1,089	65.4212	150.66	3,080.8	33,364	209.7215		0.0324150
Wood dust		76,713	563.81	6,741.1		191.4631		
Rubber dust				10.3				0.039
Metal dust		165,679	456.107					
